# Analysis of the Recomposition of Norms and Representations in the Field of Psychiatry and Mental Health in the Age of Electronic Mental Health: Qualitative Study

**DOI:** 10.2196/11665

**Published:** 2019-10-09

**Authors:** Margot Morgiève, Déborah Sebbane, Bianca De Rosario, Vincent Demassiet, Soraya Kabbaj, Xavier Briffault, Jean-Luc Roelandt

**Affiliations:** 1 World Health Organization Collaborating Centre for Research and Training in Mental Health Établissement Public de Santé Mentale Lille Metropole Lille-Hellemmes France; 2 Université Lille, Centre Hospitalier Universitaire de Lille, Pôle de psychiatrie, F-59000 Lille France; 3 Eceve, Unit 1123, Inserm Université de Paris Paris France; 4 Cermes3 Paris France

**Keywords:** ehealth, mental health, psychiatry, social representations, focus group, users, caregivers, qualitative analysis, digital tools.

## Abstract

**Background:**

For the World Health Organization, electronic health (eHealth) is seen as an effective way to improve therapeutic practices and disease prevention in health. Digital tools lead to major changes in the field of mental medicine, but specific analyses are required to understand and accompany these changes.

**Objective:**

Our objective was to highlight the positions of the different stakeholders of the mental health care system on eHealth services and tools, as well as to establish professional and user group profiles of these positions and the uses of these services.

**Methods:**

In order to acquire the opinions and expectations of different categories of people, we carried out a qualitative study based on 10 focus groups (n=70, from 3-12 people per group) composed of: general practitioners, psychiatrists, psychologists, social workers, occupational therapists, nurses, caregivers, mental health services users, user representatives, and the general public. The analyses of focus group discussions were performed independently by four investigators through a common analysis grid. The constant comparative method was adopted within this framework.

**Results:**

The interviewees expressed different problems that new technologies engender in the field of mental health. What was previously strictly under the jurisdiction of physicians now tends to be fragmented and distributed over different groups and locations. New technologies reposition care in the field of domestic, rather than therapeutic, activities, and thus the conception of care as an autonomous activity in the subject’s life is questioned. The ideal of social autonomy through technology is part of the new logic of health democracy and empowerment, which is linked to a strong, contemporary aspiration to perform. Participants emphasized that there was the potential risk of a decrease in autonomy for the digitally engaged patient, while personal empowerment could become a set of obligations.

**Conclusions:**

This qualitative research highlights the heterogeneity of opinions among the groups and within each group. It suggests that opinions on electronic mental health devices are still far from being stabilized, and that a change management process should be set up to both regulate the development and facilitate the use of these tools.

## Introduction

The field of electronic mental health (e-mental health) is particularly active and produces new tools at an extremely rapid pace [1-3], forcing people to position themselves in relation to these now unavoidable innovations that lead to major recompositions of thought [4]. Far from being a side effect or a passing fad, the development of connected objects in the mental health field is epistemologically like new approaches in psychiatry, which are based on contextually situated networks. In our opinion, this represents a fundamental trend that will nourish, and be nourished by, the already observed changes in nosographic and therapeutic categories in the field [5].

The importance of this trend is illustrated by the considerable interest in new technologies among members of the mental health field. For example, many references in the scientific literature are interested in the important potential of electronic health (eHealth) technologies for transforming and improving therapeutic and preventative health practices [6]. Not only are they likely to improve the effectiveness of care, they could also change its very nature [7]. They would thus be likely to disrupt the current methods of care, to majorly modify what we know or believe about psychiatric disorders [8], and to participate in P4 (predictive, preventive, personalized and participatory) medicine. Thus, while eHealth technologies could make it possible to improve patient-physician interactions and treatment compliance [9], they could also modify the patient-physician relationship by making it less hierarchical. At the level of the health care system, eHealth strategies could optimize the accessibility and efficiency of care, thus improving the effectiveness of treatment and reducing the cost of interventions.

The European E-men project was set up to develop an e-mental health innovation and transnational implementation platform in Northwest Europe. The project is promoting better and more accessible mental health care through the increased use of e-mental health interventions in a six-country European Union partnership. Funded by the Interreg North-West Europe Programme, the project aims to support the development and testing of electronic interventions in the different partner countries and to increase awareness about the potential of e-mental health through seminars, publications, and the development of policy recommendations. Furthermore, a cooperation platform has also been set up to address e-mental health implementation challenges in the long-term. Developing a better understanding of e-mental health acceptance is an important part of the E-men project, as it works to provide guidance on how to increase broader and more responsible implementation. Several actions have been undertaken in the E-men project to achieve such an understanding. In this paper, we will focus specifically on one of these actions, namely the analyses of the representations and declared practices of members of the field of psychiatry and mental health in the context of rapid and disruptive technological developments. We will address this question on the basis of the results of an empirical survey (the Qualitative study of m-Health expectations and uses by all stakeholders [EQUME]) conducted by the World Health Organization (WHO) Collaborating Centre of Lille (France), in the framework of its involvement in the E-men project.

## Methods

### Overview

We set up the EQUME qualitative study to collect information from the main groups of actors involved in the field of e-mental health: general practitioners (GPs), psychiatrists, mental health services users, users’ representatives (those people who are members of an association of services users or on the board of this sort of association), the public, caregivers, social workers, psychologists, occupational therapists and nurses. These groups are referred to as stakeholders throughout this paper, as they each have some connection to the e-mental health field. 10 focus groups were formed from these main groups, with a total of 70 individuals involved (Table 1). We chose the term actor because it refers to the Actor-Network Theory [10], which considers the system comprising human and nonhuman actors, including technologies.

Focus group methodology was used to collect material on topics of interest through group exchanges. The exchanges were moderated by a moderator and an assistant moderator and were the subject of audio and video recordings. It should be noted that the EQUME study was the subject of a declaration of compliance with reference methodology at the Commission nationale de l’informatique et des libertés (CNIL) (N°2040798 v 0, March 3, 2017). All participants signed a consent form to be filmed.

The focus groups were conducted in accordance with the classic criteria of this methodology, namely: (1) Six to twelve participants [11,12]; (2) meetings lasted between one-two hours [13,14]; and (3) groups were ideally led by a moderator (MM) and an assistant moderator (DS or BDR) [15]. The moderator organized the conversation by asking questions to focus the topic of conversation and by encouraging everyone to participate. To do this, a semidirective interview grid was used. The assistant was responsible for making the video recordings and creating an environment conducive to discussion.

The semidirective focus group animation grid was developed by a pluridisciplinary team (two researchers in social sciences, a psychologist, a psychiatrist, and a services user) after prior analysis of the scientific literature, based in particular on the acceptability model for eHealth devices (acceptability, usability, utility, reliability, risk) [16].

**Table 1:** Participants of the focus groups.

Categories of actors	Participants			Age of participants, n (IQR)	Knowledge of electronic mental health tools, n (IQR)
	Men	Women	Total		
GPs^a^	4	1	5	48.4 (40-59)	4.5 (3-5)
Psychiatrists	3	2	5	43.6 (25-62)	3.2 (0-8.5)
Users’ representatives	2	1	3	54.3 (29-77)	3.3 (1-6)
The public	0	6	6	38.5 (29-53)	3.2 (1-7)
Family caregivers	6	3	9	62.2 (48-74)	1.8 (0-4)
Social workers	0	5	5	43.2 (29-57)	1.6 (0-5)
Psychologist	1	6	7	35.7 (25-59)	1.7 (0-5)
Services users	11	1	12	42 (30-59)	3.7 (0-9)
Occupational therapist	2	7	9	38.4 (24-56)	1.1 (0-4)
Nurses	4	5	9	36.7 (25-48)	2.6 (0-6)
Total/Average	33	37	70	44.3 (24-77)	2.2 (0-9)

^a^GPs: General Practitioners

### Data Analysis

To analyze the exchanges of the 10 focus groups, a thematic analysis grid was developed. From the first video recorded, the pluridisciplinary team of researchers independently created a list of the mentioned topics and categorized them. These initial categorizations were then pooled to form the analysis grid that was then applied to all groups. Each focus group was the subject of two independent analyses by two researchers, and then information was pooled during harmonization meetings. Disagreements on categorization were settled by a discussion using the description associated with each category. This method produced five major themes: (1) relationship patterns and tensions between psychiatry and mental health; (2) distribution of skills or methods of collaboration between Information and Communications Technology (ICT) and health professionals; (3) impact of eHealth on the caregiver and patient relationship; (4) process of autonomization; and (5) regulation of the sociotechnological ecosystem. Within these five major themes, the different positions of the actors in each group were recorded, with most participants involved in the discussions. We used a constant comparison analyses methodology, such as proposed by Glaser [17], a methodology that is well suited to analyze multiple focus groups [12].

## Results

### Overview

We have identified 5 main themes, divided into 19 subthemes. The first theme was related to “Relationship Patterns and Tensions Between Psychiatry and Mental Health,” which was divided into 4 subthemes: (1) psychiatry versus mental health; (2) psychiatry and mental health: a hierarchical reversal; (3) from psychiatry to mental health: no paradigm shift; and (4) from mental health to mental disability: a shift from a curative to a rehabilitative approach.

The second one was related to “Distribution of Skills or Methods of Collaboration Between Information and Communications Technology and Health Professionals,” which contained 4 subthemes: (1) the impossibility of replacing human actors with technology; (2) the possibility of replacing human actors with technology; (3) collaboration between ICT and health professionals; and (4) technology finely integrated into everyday life.

The third main theme was associated with the “Impact of Electronic Health on the Caregiver and Patient Relationship,” and had three themes: (1) technology as an agent of change and improving connections; (2) electronic mental health is a barrier to the health care relationship; and (3) electronic mental health only brings about changes without a paradigm shift.

The 4th theme, relating to the “Process of Autonomization,” was divided into 5 subthemes: (1) technology participates in processes of expertise and empowerment; (2) in favor of maintaining the caregivers’ monopoly; (3) developing technological habits marked by hyperreflexivity and dependence; (4) contemporary aspiration for individualism and to perform; and (5) social injunction to autonomy.

Finally, the last main theme that emerged from the analysis was the “Regulation of the Sociotechnological Ecosystem,” dealing specifically with regulation under the authority of the health system, the lack of a therapeutic framework leading to an extension from the private to the public domain, and self-regulation of the electronic health ecosystem.

### Relationship Patterns and Tensions Between Psychiatry and Mental Health

#### Summary

The field of mental health is marked by numerous conceptual, organizational, and legislative developments, including developments in e-mental health that question and often upset the relationship between health and mental health in terms of normal, pathological, individual, and systemic mental health, and in terms of the limits of the field of psychiatry. Four main positions about the field stand out among the groups interviewed.

#### Psychiatry Versus Mental Health

For GPs, social workers, users’ representatives and the public, mental health cannot be superimposed on psychiatry. For GPs, the severity of the disorders set up the boundaries and territories of expertise and power of two types of professionals: psychiatrists and psychologists. Thus, severe disorders were under the jurisdiction of physicians and fell within psychiatry, while mild disorders were under the jurisdiction of psychologists and fell within the field of mental health. The users’ representatives expressed that same idea and specified that the bounds of the field of psychiatry lie in public intervention, while those of mental health lie in private care. They added that the mental health and psychiatry dyad is usually viewed positively on the mental health side while the psychiatric side has negative connotations and is often associated with confinement.

For the public, mental health would be the social, mental, and subjective topic while psychiatry would be the natural, cerebral, and scientific subject. Philosophers and anthropologists described a movement that began in the 1980s to value naturalistic explanatory models (the cerebral etiology of the disease), which is associated with the advent of neuroscience [18]. While the first model focuses on the enhancement of the individual’s life and addresses a socialized subject, the second, biological and cognitivist perspective addresses a natural or cerebral subject [19]. In this naturalistic model, the symptoms of the disorder or disease are emptied of their personal and moral contents, and all that is left is the biological and cerebral science. This depsychologization strategy is classically considered guilt-free [20], which could explain its prevalence in the public, but it must be handled with caution. For example, it is not necessarily preferable in society to be have a brain injury than to have been diagnosed with a psychological disorder. The hope is that the advent of ICT could potentially update the debates linked to the renaturalization of disorders and their related stigmatization issues by basing their management around technology designed specifically for that purpose.

Social workers and the public stated that, “mental health is (psychic) well-being.” They also added that wellbeing was not a subdomain of health. According to the public, it was up to the legislator to define the contours of what is considered or not to be health:

Health issues are not treated in the same way as wellness issues.

Legislation exists.

It is up to the High Authority for Health (HAS) to say what is health and what is not.

Social workers and the public did not seem to support the process of extending the pathology of a disease to symptoms that were not originally covered by it. This distinction may appear in contradiction with the general dynamics of recognition of health as a medical problem, and the tendency of medicine to include wellbeing in its field, as noted by Ehrenberg. Referring to the notion of mental health, the sociologist specifies that:

Taking charge of schizophrenia or improving its performance and psychological balance, in work, sexuality or relationships with children fall under the same label. Mixing frankly pathological problems with concerns for well-being, the notion is so broad that it is indeterminate [21].

#### Psychiatry and Mental Health: A Hierarchical Reversal

Psychologists, psychiatrists, some of the services users, and some of the occupational therapists interviewed considered mental health to be a broader field that includes psychiatry. For occupational therapists, the term mental health was more generic, inclusive, and extensive and was focused on prevention in public health, while psychiatry was synonymous with illness and pathology. Psychiatrists argued that they must not bear sole responsibility for the field of mental health. For psychologists and certain users:

mental health concerns the individual, the person, it contains the individual’s lifestyle, well-being and social relations.

This is illustrated by Ehrenberg’s theory of a hierarchical reversal:

mental illness is now an aspect of mental health. The madman to be locked up is only one element in a larger whole which has encompassed him, that of the citizen in difficulty who must be supported (but also repressed, contained differently than in the past) and who must be the actor of his disease [21].

This process questions the continuum between the patient and those who are healthy, as well as the concept of them and us, which is part of an evolution of representations and practices that has occurred over decades. This evolution went from a paternalistic view of care in the 1950s, to a patient-centered view in the 1990s, to a collaborative and partnership-based system of care beginning in the 2010s, leading to the normativity of full inclusion [22].

#### From Psychiatry to Mental Health: No Paradigm Shift

For nurses, some of the services users and some of the occupational therapists interviewed, mental health and psychiatry were synonymous terms, as “it’s just a matter of words.” The only development reported concerns communication and image. One nurse said:

Mental health is less scary than psychiatry, but I don’t see a paradigm shift with the name change: it’s just sweeter to hear.

One occupational therapist also said that mental health is “the new buzzword” to make psychiatry less stigmatizing. Mental health would therefore strictly cover the field of psychiatry, but the use of the term mental health could potentially reduce stigmatic representations associated with psychiatry.

#### From Mental Health to Mental Disability: A Shift From a Curative to a Rehabilitative Approach

Caregivers were categorical, stating that health is “when it goes well.” No caregiver appreciates or uses the term mental health and they instead use the idiom “psychic disability.” This logic of “disabling situations” [23] is no longer only interested in the causes but also in the consequences of health problems in a given environment [24]. This new logic of disability not only inserts mental pathology into a broader frame of reference than illness, but also changes its meaning [25]. Morgiève et al state that:

In the shift from the patient with a psychiatric-neurological illness to the ‘person with a disability,’ the medical objective of reducing or eliminating symptoms loses its centrality. It becomes one of the elements of a system aimed at reducing the impact of these symptoms on daily life in order to improve the quality of life [26].

A shift thus takes place from a curative logic inscribed in a health model (eg, the problem is only individual, is based on an anomaly, is a matter for specialists) to a rehabilitative logic inscribed in a social model (eg, the problem is also in the social structure, is based on differences, is a public question) [23].

### Distribution of Skills or Methods of Collaboration Between Information and Communications Technology and Health Professionals

#### Summary

The introduction of ICT in the field of mental health brings the question of the distribution of skills, the fields of jurisdiction, and the methods of collaboration between ICT and health professionals. Four means of collaboration were proposed by the different groups.

#### Impossibility of Replacing Human Actors with Technology

For most participants, technology could not replace health professionals. Different representations are associated with this impossibility. Some GPs legitimized and defended their field of work by reaffirming their influence while discrediting the ICT field:

When I hear e-health, I still think it’s about health at discount prices and fashionable gimmicks, something commercial; I don’t feel like we’re talking about medicine, medicine is about serious stuff you use.

User representatives agreed that “it’s just a gadget.” According to this group, mental health would be the territory of medicine and under the authority of the doctor who guarantees its seriousness, so technology for managing it would be reduced to an accessory status. Psychologists wished to confine the role of eHealth to that of a therapeutic adjuvant because they feared “that someone will steal know-how that could be duplicated by the machine.” Nurses, who recognized a possible superiority of technology, feared that this could have consequences on the employment of health professionals. This fear was more generally found among respondents of a survey conducted by the European Commission. Its results showed that respondents were pessimistic about the impact of robots and artificial intelligence on jobs, and more than 7/10 thought they steal jobs and cause more job loss than job creation [27].

For services users, caregivers, and the public, technology could not replace the health professional but could be complementary. All insisted on the need for it to be placed under medical authority:

removing the human is complicated; digital tools must just accompany doctors, patients, not replace medicine, especially in mental health.

Nurses stressed the centrality of the human and narrative dimensions in care, which they felt could not be supported by ICT:

it is not desirable that an e-mental health tool replace a professional in diagnosis because a patient is also his history.

For occupational therapists, tools like ICT sped up time “when you have to take it in psychiatry.” GPs and users’ representatives placed emphasis on the importance of communication. The first group asserted that psychiatry is care through communication and “therefore incompatible with e-health,” and the second said that eHealth “is not real health, the one where we talk with patients because health without a third party is not health,” while patients who used it “believe that they can heal themselves with their mobile phone.” One user representative concluded that “it’s discounted health.” Psychologists also mentioned the impossibility for ICT to replace social interaction, but more specifically the transfer that remains strictly under their jurisdiction. They nevertheless thought that these new tools would redefine their roles.

#### Possibility of Replacing Humans with Technology

For social workers, the process of replacing the health professional with technology “is in progress,” while for some psychiatrists it was still only a “high probability,” and for nurses “the technology is able to do instead and do better.” Some psychiatrists spoke about the possibility of a future superiority of ICT over humans, which is associated with fears of dehumanization and of the impossibility for physicians to regulate the use of technological devices.

#### Collaboration Between Information and Communication Technology and Health Professionals

Some participants from the public envisaged that in the future, “if knowledge grows, we can have devices as capable as a doctor.” Some psychiatrists spoke about the future superiority of the human and machine pairing over the human alone. In these two configurations there is no opposition between these sociotechnological groups but rather a collaboration between human and machine.

#### Technology Finely Integrated Into Everyday Life

GPs considered that wanting to make eHealth its own, distinct field was nonsense. According to these doctors, there would be no tension related to the use of these tools that they described as already being a part of everyday life. This inclusion of ICT in normal activity seems to extend from therapeutic activities to domestic activities, blurring the boundaries that once separated them. The conception of care as an autonomous activity in the subject’s life was thus questioned in the public group, with an example of “Is the jogging tracker a mental health tool?”

### Impact of Electronic Health on the Caregiver and Patient Relationship

#### Summary

The introduction of ICT into the therapeutic health care relationship modifies previously established regulatory procedures, thus forcing patients and physicians to renegotiate the rules they would normally follow. The impact on the patient and caregiver relationship caused by eHealth is organized along three axes.

#### Technology as an Agent of Change and Improving Connections

For most participants, e-mental health made it possible to find a therapeutic relationship and was a means of connecting to others. Participants from the public stated that “we think that it tends to distance us” whereas “just the fact of making the effort to send a message, the link is stronger.” The public nevertheless considered this potential improvement in connection with others to be based on the acceptability of the technology by users. Caregivers emphasized the reciprocity and bidirectionality of these tools, which are a way for the patient to come into contact with professionals for the first time but also a way for health professionals to come into contact with patients through tools they use on a daily basis. Psychiatrists mentioned situations where patients “come to show their applications on smartphones.” New technological tools chosen by the patient thus intervene in the therapeutic relationship, leading to a redistribution of the roles of each of the actors. Psychologists believed that eHealth gave a new social place to patients who “re-enter in the society.” These tools make it possible to imagine new projects whose concrete implementation makes it possible to “change the team’s view of the patient.” Psychologists also noticed the equal spread of these tools, thanks to which “everyone will have their little coach in their pocket,” and their capacity for catering to each individual because each one will have “his application according to the situation.”

One psychologist said that, “you will call your psychiatrist into your living room.” This scene illustrates a strong shift in the paradigm, as the patient becomes the one who brings the doctor to them and into their home. Thus, ICT seems to reconfigure hierarchical relationships and locate care not only in the therapeutic field but also in the private sphere. If there were no longer any boundaries delineating the field of health from the rest of life, psychologists questioned the therapeutic dimension of this continuum:

but can we intervene in the patient’s life all the time, is it therapeutic?

Caregivers were interested in the possibility of patients questioning the medical profession:

I will be happy when health professionals will be afraid of the note their patients will put on the internet.

Users also evoked a possible extension of their power generated by the use of e-mental health devices:

It allows for discussion with the doctor, without being superior but being better informed. The relationship is better.

#### Electronic Mental Health is a Barrier to the Health Care Relationship

For occupational therapists, e-mental health devices could be a barrier to access to care. They said that they fear that these tools discouraged people from consulting professionals, or even lead them to self-diagnosis. According to them, ICT lead to a damaging lack of “communication and interpersonal relationships“ and it would even be a means for physicians to “get rid of their patients.” For some GPs, eHealth could hinder the therapeutic relationship. A nurse reported his patients saying, “I stopped my treatment because on the internet...” and he then concluded that eHealth “serves to make people sicker.”

#### Electronic Mental Health Only Brings About Changes Without a Paradigm Shift

For most nurses and user representatives, and some GPs, eHealth tools were only new ways to practice old techniques and did not create a new paradigm for patient management. One doctor specified:

the maniac depressed [outdated term] he uses paper, the bipolar [current term] he uses his computer.

One user representative illustrated the lack of relational change by saying:

You can’t be friends on Facebook with your psychiatrist.

According to them, the technological space did not entail any modification in the health care relationship.

### Process of Autonomization

#### Summary

The participants’ positions on the roles of new technologies in terms of individual empowerment and empowerment processes can fit into five categories.

#### Technology Participates in Processes of Expertise and Empowerment

For the public, social workers, services users, and nurses, eHealth was changing the role of the patient and making them a more active and autonomous actor. Mental health services users explained that the new technologies allowed them to access more information and that “the fact of having information makes us independent of a doctor.” According to nurses, e-mental health allowed for “shared responsibility between caregiver and patient” and thus that “we leave the paternalistic model” behind. GPs emphasized the disruptive dimension of ICT in mental health that allowed “a new paradigm, a new system, a new use impossible to do otherwise.” According to a general practitioner, this process could even bring legitimacy and competence to patients in a field that would no longer be exclusively, or even at all, that of doctors.

#### In Favor of Maintaining the Caregivers’ Monopoly

Some psychologists and caregivers saw the potential for eHealth to help or empower patients. However, they made it an essential condition “that it passes through the human,” that the tools were used as a secondary accessory, and that it was validated by health professionals and not by patients. For all occupational therapists, eHealth reduced individual empowerment because the patient did not know what was good for them or what they should do with the information they found. In the discourse of these three groups, patients seemed to be considered passive targets who were supposed to comply with the prescriptions of health professionals who possessed the legitimate knowledge of their condition and the best way to treat it [28-30].

#### Developing Technological Habits Marked by Hyperreflexivity and Dependence

For some psychologists, occupational therapists, and social workers, the patient became dependent on a machine:

I don’t think it gives autonomy, it organizes rather dependence on the device.

Psychologists described these tools as invasive and that they needed to be used less often. ICT in mental health can help one develop some level of scientific expertise and a critical awareness of one’s activities, but it also develops inward-looking attitudes and hyperreflexivity. For caregivers and nurses, ICT did not increase the reflexivity of patients who, because of their pathology, would have already developed a propensity for too much self-observation:

it is in the normal process of illness to seek to rationalize, to focus, to question, to talk about oneself in the end.

#### Contemporary Aspiration for Individualism and to Perform

For some psychologists:

the omnipresence of a health medium permanently in one’s pocket is likely to develop a personal emulation to do better, to want better, and to be healthier.

Users described eHealth as individualistic, as “it’s every man for him.” Electronic health seems to correspond to a strong contemporary aspiration to perform as well as to an ideology of individualism and taking control of one’s life. Taking responsibility for one’s life as an individual, rational actor is thus privileged and promoted in contemporary industrial societies [31].

#### Social Injunction to Autonomy

User representatives were critical:

Empowerment with technology is a complete fake.

Empowerment / ICT / Mental health = bad stuff they want to impose on us. The philosophy of regaining power over one’s life is good but it is quoted in all government reports, it seems confusing.

Patient engagement in health care is at the forefront of research policy and practice and is now widely recognized as an essential ingredient of a high-quality health system. However, the discourses of the digitalized and digitally engaged patient are seen as part of a left-wing policy orientation in care. These discourses position patients as ready to actively engage in their own health care and promote their own health, which may be seen as an attempt to shift the burden of responsibility from the State to the individual [28]. In the discourse of the digitally engaged patient, individual empowerment becomes a set of obligations that they need to take care of themselves [29]. There is then a paradigm shift from “my health is my doctor’s responsibility” to “my health is my responsibility and I have the tools to manage it” [32].

### Regulation of the Sociotechnological Ecosystem

#### Summary

Because of the way digital data is created, stored, and used, both personal and private practices are quickly interwoven within networks and economies [33]. The means of regulating the mechanisms of this new sociotechnological ecosystem are therefore essential, since private practices are caught up in a collective system in which different actors compete for control of the field [34]. For this theme, three types of views emerged from the focus groups.

#### Regulation Under the Authority of the Health System

Some GPs, social workers, and nurses affirmed the need for regulation to guarantee the reliability, security, and confidentiality of computerized health data. However, a GP explained that technological devices could guarantee more data security than some “archaic” tools. For users’ representatives, the use of ICT “enters a health system and a health system has its rules.” Psychologists specified that validation of tools must be done by health professionals and not by patients, while psychiatrists were concerned about the inability of their professional group to regulate their use. Users mentioned the Ministry of Justice, the Ministry of Health, researchers, doctors, professionals who represent patients (ie, families and peer-helpers), as legitimate bodies and actors who could regulate the field of eHealth. Some GPs and nurses were also concerned about the extension of their legal liability. They expressed fear of being watched and judged, and of potential legal risks. A general practitioner thus pleaded for a “presumption of benevolence” towards the medical profession.

#### Lack of a Therapeutic Framework Leading to an Extension From the Private to the Public Domain

Occupational therapists and some participants from the public, as well as some caregivers and some users’ representatives, described a current lack of a therapeutic framework in eHealth that seems dangerous to them as, “we risk being passively invaded.” Some caregivers pointed to the need for standards and regulatory systems. The public also raised the question of the confidence that can be placed in the technological tool. To guarantee the safety of users, it seemed essential to them to be able to identify three types of groups: those who hide behind the development of the tools, those who have a financial interest, and the people “behind the computer from whom medical advice is sought.” These participants were aware of the complexity and multitude of human and nonhuman actors working together to configure these devices and the need to understand how users, designers, developers, and funders were able to construct, interpret. and negotiate the generated data.

One user representative worried:

mental health is in your head, new technologies are open so you open your head open.

Another user representative stated that eHealth could not contribute to individual empowerment because the implementation of technological tools required very complex settings and that “it therefore becomes a collective affair.”

These comments echoed various analyses that say that the human network is contributing to a transformation, initiated over the last thirty years, in which individual subjectivity has become a collective issue [25]. The agency of each individual now seems to be at the center of social life:

the more the individual is considered as an autonomous whole, which must be able to decide and act by himself, the more the question of his interiority becomes a public concern [21].

#### Self-Regulation of the Electronic Health Ecosystem

Some caregivers, users’ representatives and members of the public believed that the eHealth ecosystem is self-regulating. According to one user representative, users of technological devices were “health actors” and he saw “no risk of capture.” The use of eHealth would thus result from free will, where “everyone sets his limits.” Participants from the public insisted on the concept of ethical and legal acceptability of these new technologies, which according to them continued to prevail.

These results are all summarized in Table 2.

**Table 2:** Synthesis of the results.

Discussion themes and opinions of stakeholders		GPs^a^	Psychiatrists	Service users’ representatives	The public	Caregivers	Social workers	Psychologists	Services users	Occupational therapists	Nurses
**Relationship patterns and tensions between psychiatry and mental health**											
	Psychiatry versus mental health	✓		✓	✓		✓				
	Psychiatry and mental health: a hierarchical reversal		✓					✓	✓		
	From psychiatry to mental health: no paradigm shift								✓	✓	✓
	From mental health to mental disability: a shift from a curative to a rehabilitative approach					✓					
**Distribution of skills or methods of collaboration between information and communications technology and health professionals**											
	Impossibility of replacing human professionals with technology	✓		✓	✓	✓		✓	✓	✓	
	Possibility of replacing humans with technology		✓				✓				✓
	Collaboration between information and communication technology and health professionals		✓		✓						
	Technology finely integrated into everyday life	✓									
**Impact of electronic health on the caregiver and patient relationship**											
	Technology as an agent of change and improving connections	✓	✓		✓	✓	✓	✓	✓		✓
	Electronic mental health is a barrier to the health care relationship	✓								✓	✓
	Electronic mental health only brings about changes without a paradigm shift	✓		✓							✓
**Process of autonomization**											
	Technology participates in processes of expertise and empowerment	✓	✓		✓		✓		✓		✓
	In favor of maintaining the caregivers’ monopoly		✓			✓		✓		✓	
	Developing technological habits marked by hyperreflexivity and dependence						✓	✓		✓	
	Contemporary aspiration for individualism and to perform			✓				✓			
	Social injunction to autonomy			✓							
**Regulation of the sociotechnological ecosystem**											
	Regulation under the authority of the health system	✓	✓	✓			✓	✓	✓		✓
	Lack of a therapeutic framework leading to an extension from the private to the public domain			✓	✓	✓				✓	
	Self-regulation of the electronic health ecosystem			✓	✓	✓					

^a^GPs: general practitioners

## Discussion

### Primary Findings

The interviewees expressed different problems that new technologies engender in the field of mental health. What was previously strictly under the jurisdiction of physicians now tends to be fragmented and distributed over different groups and locations. New technologies reposition care in the field of domestic, rather than therapeutic, activities, and thus the conception of care as an autonomous activity in the subject’s life is questioned. The ideal of social autonomy through technology is part of the new logic of health democracy and empowerment, which is linked to a strong, contemporary aspiration to perform. Participants emphasized that there was the potential risk of a decrease in autonomy for the digitally engaged patient, while personal empowerment could become a set of obligations.

This qualitative research highlights the heterogeneity of opinions among the groups and within each group. It suggests that opinions on electronic mental health devices are still far from being stabilized, and that a change management process should be set up to both regulate the development and facilitate the use of these tools.

### Limitations

The aim of this study was to understand some of the existing representations and concerns of the main groups affected by the use of e-mental health tools. The study did not claim to be exhaustive or even representative of such groups, and data obtained are essentially representative of two major, urban, French cities. Moreover, two specific problems must be mentioned. First, four people among the users’ representatives group did not show up or cancelled their participation in the focus group too late to be replaced, so users’ representatives are thus underrepresented in the results. Second, the system users’ group is composed of an unexpectedly high proportion of men and the potential reasons for this bias are unknown.

### Conclusion

The data we have presented highlights an important inter- and intragroup, and even intraindividual, fragmentation of points of view on eHealth, with participants making statements that may appear contradictory with each other. This suggests that positions on these new technological devices are still far from being stabilized and may evolve even during a focus group. Indeed, these devices themselves are a very recent innovation, are little known, and evolve very rapidly and unpredictably.

Another apparent result is that, far from allowing the cooperation between actors that they are supposed to promote, the emergence of these apparently diverse reactive mechanisms, classical defensive tensions, and positioning within psychiatry and medicine in France have generated groups of actors who want to defend their categorical interests and their social or socio-professional identity. The existence of these reactions leads to the hypothesis that there is concern among participants about observable and imaginable changes generated by the development of ICT in the health field, and also suggests that a specific change support process must be put in place to allow good ownership and optimal use by stakeholders. The specificity of these new tools is that they enable the overcoming of the traditional boundaries and modes of regulation and communication, which has allowed the move towards the fluid and evolving functioning of individuals in a network.

This study must be seen as a first step toward a more detailed understanding of the current representations in e-mental health. A quantitative study concerning services users is already ongoing in the framework of the EQUME study. Studies about actual health care practices in the field are also needed to complement this first study on representations, as well as more organizational studies concerning change management support for the French e-mental health ecosystem.

## Abbreviations

CNIL: Commission Nationale de l’Informatique et des Libertés
eHealth: electronic health
e-mental health: electronic mental health
EQUME: Qualitative study of m-Health expectations and uses by all stakeholders
GCS: Groupement de coopération sanitaire
GP: general practitioner
ICT: Information and Communications Technology
UNAFAM: Union nationale de familles et amis de personnes malades ou handicapées psychiques
WHO: World Health Organization
